# The Milch Goat

**Published:** 1918-11

**Authors:** 


### The Milch Goat.

The present high cost of milk and dairy products generally is not entirely a war condition, nor due to any single and conspicuous factor. While subject to explanation in regard to various details, it is not due to any considerable recent increase of density of population, to a shortage or vitiation of grazing land, to any increase of disease among cattle, to any marked competitive factor which should tend to distract agricultural labor from milk production. Two factors likely to be overlooked are of importance: Cleanliness in handling dairy products, as in any other particular, costs time, brains and therefore money, although we are slowly learning this fact and still are inclined to regard cleanliness as an additional beenfit which can be had for nothing but the desire. The dairy business has, gradually but on the whole generally and almost completely, become big business instead of a scattered retail one. This ought, theoretically, to tend to economy and cheapness but, as a fact which we are equally slow to realize, it practically always means the reverse. The essential reason why milk and its derivatives have increased in price is that the men producing them, want more pay—like almost every one else. The only way to preserve some measure of cheapness in these vitally necessary foods is to attempt the fulfillment of the politician's highly popular slogan—High wages and low prices! Whether success is possible is another question.

Meantime, the goat offers itself as a possible solution of the immediate problem for a considerable part of the population interested, even in cities. The average of a large herd yields about a quart and a half of milk a day, permanently, including inactive does and the necessary bucks. During the milch period of somewhere about 3-5 months, the per capita yield is about a gallon a day, sometimes more. Co-operation in small family groups would obviously yield an adequate supply, except for the impossibility of arranging a schedule free from fluctuations above and below the required amount. The goat is, so to speak, a portable animal. It can literally be transported to care for the needs of children, and is commonly driven from house to house to deliver fresh milk, in those continental cities where it is a staple source of milk. It is portable in the less direct but more practical sense of being available where a large animal like the cow would be entirely impracticable. Its intestinal physiology is also a practical advantage. Its upkeep is relatively low, though it is scarcely necessary to say that it does not nourish itself on entirely inedible substances. It can be kept in a small back yard, is not especially objectionable in a city, can be housed in a dry-goods box or two piano boxes joined or in a shack that any boy can construct without appreciable expense. It can be kept clean by turning the hose on it in the summer and, even in winter, as readily as a large dog.

Goat milk is not of a disagreeable flavor as popularly supposed but is of approximately the composition and taste of good cow's milk. The goat is practically immune to tuberculosis and the only appreciable sanitary danger, eliminated by reasonable and obvious precautions, is in regard to the importation of Malta fever. Whether the immunity to tuberculosis has more than a negative value in regard to the use of milk for human beings and especially infants and children, is not yet decided.

The principal practical obstacles to the immediate use of goats as milk producers are: conflict with health ordinances, the liability of the retail user to come up against fancy prices either as a purchaser of milk, of a goat or its feed, according to well known economic principles or lack of principle, as for anything that is new and not standardized by long business custom. The individual labor necessary must also be considered. Mr. W. C. Bull of this city is an authority on goats and it seems to us that the time has come when a serious attempt should be made to solve the milk problem by the use of the goat.

Note: Grade B, Pasteurized Milk, retails at 10 or 11 cents in N. Y. City; at 14 cents in Buffalo.

